# An instrument as an action against the blind spot of acute medical care in general practice - a systematic review

**DOI:** 10.1186/s12875-025-02749-6

**Published:** 2025-03-08

**Authors:** Johannes Rieken, Daniel Hötker, Christoph Strumann, Jost Steinhäuser

**Affiliations:** 1 Institute of Family Medicine, University Medical Center Schleswig-Holstein, Campus Lübeck, Ratzeburger Allee 160, 23538 Lübeck, Germany; 2 Bundeswehrkrankenhaus, Hamburg, Germany

**Keywords:** Urgent medical cases, General practitioners, Primary care, Systematic review, Instrument, Acute care, Emergency care

## Abstract

**Background:**

Increasing visits to out-of-hours practices and Emergency Departments (EDs) for non-life-threatening urgent cases (NLTUCs) have placed a significant burden on healthcare systems worldwide. General practitioners (GPs), as the first point of contact in primary care, play a critical role in managing acute medical cases. However, limited research has focused on their contribution to acute care, and tools for assessing these cases remain non-existent.

**Aim:**

This review aimed to identify instruments for detecting acute medical cases in GP practices, addressing the gap in tools and frameworks specific to the primary care setting.

**Methods:**

A systematic review was conducted following PRISMA guidelines. Searches were performed in PubMed, CINAHL, Scopus, and Web of Science, focusing on studies describing instruments for acute care assessment in primary care.

**Results:**

Of 1,560 identified studies, one met the inclusion criteria. The included study described a coding tool designed to assess the complexity of GP consultations, using the ICPC-2 classification system. While this tool effectively captures the multifaceted nature of GP encounters, it was not specifically designed to measure urgency in acute care.

**Discussion:**

The review highlights a significant gap in tools for assessing urgency in GP practices, contrasting with established hospital triage systems. Adapting existing tools to incorporate urgency assessment could illuminate the critical impact of GPs on reducing ED burden and managing acute cases.

**Conclusion:**

The identified tool for assessing consultation complexity could be adapted to evaluate urgency, highlighting the critical yet underrecognized role of GPs in acute care.

**Supplementary Information:**

The online version contains supplementary material available at 10.1186/s12875-025-02749-6.

## Background

Patients with none-life-threatening urgent cases (NLTUCs) put a strain on medical systems, especially at times, when general practitioners’ (GPs) practices are closed [1]. These patients have a prominent space in political discussions and scientific literature [2, 3]. With the numbers in emergency patients rising, the increasing pressure on medical institutions is challenging the political agenda [4].

At the same time life-threatening urgent cases (LTCUs) that do not appear in any form of discussion or political agenda are treated competently and safely every day in GP practices.

Visits to out-of-hours practices as well as self-admissions to the Emergency Department (ED) have increased steadily over recent years [5] challenging health care systems worldwide [6]. A number of solution approaches have been suggested based on research in the hospital setting [6], although the majority of all medical cases (defined as a patient encounter where a healthcare professional assesses, diagnoses, and manages a health concern) worldwide are exclusively dealt with in the primary care setting [7].

The absence of published data on the amount and types of acute medical cases exclusively cared for in primary care cannot be explained by their non-appearance in GP practices. Yet these cases remain invisible leading to hospital focused attention in decision makers. In Germany, GPs make up about 36% of the doctors working in the ambulatory setting [8].

In contrast to the hospital sector, where assessment tools such as triage systems for emergency rooms are established and well researched [9], there is a lack of research on acute care provision in the primary care setting. As a consequence, little is known about the contribution of GPs in treating urgent medical cases, despite the fact that they are typically the first point of contact in primary care [9].

The care provided by general medicine is holistic and the patients are often complex [10]. GPs deal with any health related problems regardless of gender or age [11]. Their role includes addressing early-stage, undifferentiated illnesses requiring acute care, treatment and management of chronic medical problems and essential areas of prevention and rehabilitation [12].

Issues addressed by GP`s cannot be limited to the mere subject of medical inquiries. As a central institution in the community, GPs deal with matters of physical, psychological, social, cultural and environmental dimensions [11]. They view patients individually in the context of their families or social environments, also in the context of the patient’s own home. GPs often treat their patients for decades. This longitudinal aspect of the doctor-patient relationship is a fundamental principle of general medicine and is based on the wide range of responsibilities the GP has for her or his patients [12].

The organization of GP care varies widely across countries, influenced by healthcare funding models, regulatory frameworks, and cultural practices. For instance, Australia and the United Kingdom have well-established after-hours GP services integrated with urgent care clinics, whereas in Germany, after-hours care relies heavily on regional on-call systems [13]. After-hours GP care in Germany operates through a structured network managed by the Associations of Statutory Health Insurance Physicians, featuring regional on-call systems and on-call service clinics. These services address a wide range of cases, from urgent to non-urgent, ensuring accessible primary care outside regular hours with the intention of reducing the strain on EDs [14]. In some regions, GPs collaborate with hospitals [15]. Data on medical cases from various institutions is often extracted from medical documentation, which includes codes describing diagnoses, reasons for encounters, or medical procedures [16, 17]. The International Statistical Classification of Diseases and Related Health Problems (ICD-10) is the most commonly used coding system for medical diagnosis in the world [16]. However, it is an instrument designed for measuring mortality to allow comparisons between hospitals and countries.

The International Classification of Primary Care (ICPC) is a coding system, specifically designed to code reasons for encounter in a GP practice’s setting. It was developed by the World organization of Family Doctors (WONCA) International Classification Committee (WICC) and introduced in 1987 [18]. The revised ICPC-2 was introduced in 1998, accepted into the World Health Organization’s Family of International Classification and is in active use in a number of countries as the standard coding system for GPs, e.g. Portugal and Brazil. This classification system is able to code information on patient care from the initial RFEs to the final diagnosis, making it more patient-centered and therefore more useful in the context of general medicine. Since 2020, the reformed ICPC-3 is available and is currently being implemented in several countries.

In many countries i.e. Germany, the ICPC is used in research purposes only and therefore not used as a coding tool in GP practices [19].

Given the prominent role of GPs as the first point of contact for a wide range of complex health problems on the one site and the lack of knowledge about their contribution in treating acute medical cases on the other, this review aimed to identify existing instruments for measuring acute medical cases in GP practices.

## Methods

### Design and search strategy

This systematic review was conducted following PRISMA guidelines. The search strategy encompassed electronic databases, including PubMed, CINAHL, Scopus, and Web of Science, to ensure comprehensive coverage of relevant literature. The initial search was performed in March 2024. The search protocol was not registered and not published. It is attached in the appendix (Attachment A).

### Inclusion and exclusion criteria

Studies were included if they described instruments for measuring acute medical cases in GP clinics, regardless of study design or publication date. Only studies in English or German were considered.

To ensure relevance to the primary care setting, studies focusing on instruments for acute medical cases in other clinical contexts (e.g., EDs, intensive care units) or specific medical entities (e.g., asthma, anaphylaxis) were excluded.

### Screening

The screening process followed a two-step approach: First a title-abstract screening was performed followed by a full-text screening of the studies identified as eligible based on the inclusion and exclusion criteria. Two independent researchers (JR, DH) performed the screening. Any conflict was resolved by discussion and the intervention of a third reviewer (JS). After achieving consensus, the remaining publications were either included or excluded.

The screening process was facilitated using the Rayyan web tool [20].

### Data extraction and assessment

Extracted data included the following information on the development, validation, and application of the identified instruments. The quality of studies was assessed by two reviewers using the Cochrane ‚Risk of Bias‘-Tool [15]. One reviewer extracted the data from the included studies and the second reviewer checked the extracted data. Disagreements were resolved by discussion between the three reviewers with the aim of reaching a consensus. Data were extracted into standardized tables that included author, publication year, study design, participants, interventions, setting, outcomes, measurements and main findings.

### Ethical consideration

Since the systematic research was entirely focused on previously published literature and did not in any way include human participants, approval by an institutional review board was not required.

## Results

### Study selection

The database searches resulted in a total of 1560 studies. After removing 268 duplicates, the titles and abstracts of 1292 of the publications were screened.

Based on the inclusion and exclusion criteria 17 full texts of the publications were reviewed, resulting in one study being included.

### Included publication

The included British study, published by Procter et al. in BMC Family Practice in 2014, is titled “Complex consultations in primary care: a tool for assessing the range of health problems and issues addressed in general practice consultations.” [21].

It is based on observational data without randomized controls.

The objective of the study included was to develop a tool that can be used to measure the number and type of problems discussed in primary care consultations.

The researchers developed a coding proforma that can be used to code the reasons for a patient’s consultation of a GP. Each problem that a patient presents can be identified using the ICPC-2 code and can further be subcategorized into different issues using the proformas issue type definitions. These issue types cover a vast number of aspects that a problem presented to a GP may content and reach from physical, emotional, social, administrative, medication related over behavioral or medicalized health prevention to third party issues.

The tool is formatted as a table that can be printed out or filled in digitally. Details of the problems discussed will be noted in one column. Adjacent columns collect data on the ICPC-Code, the person who brought up the problem, the different types of issues and whether or not the problem can be found in the doctor’s notes (represented in readcode or as a written down diagnosis).

The study concluded that the final coding proforma could effectively record the different problems raised in a consultation as well as the different dimensions (issues) of each problem. The tool provides a comprehensive framework for understanding the multifaceted nature of GP consultations.

The primary strengths of the included study are the thorough development process for the coding tool, the assessment of inter-rater reliability, and the comprehensive reporting of methods and results. The main limitations are related to performance bias, as the participants were aware they were being recorded, which could influence their behavior. However, this does not significantly undermine the study’s main objective of developing and validating a coding tool for assessing the complexity of primary care consultations.

## Discussion

This review’s aim was to identify instruments for measuring acute care in GP consultations. The identified study did not specifically focus on acute care but more on the complexity of consultations in general. This finding affirms a significant gap regarding the number of instruments to assess acute care in primary care compared to the hospital setting [22, 23].

The various levels of urgency in medical cases GPs encounter every day have not been assessed yet because cases are so diverse and complex [24]. Measuring urgency in a setting designed solely for the purpose of treating acute medical cases is an obvious undertaking. The urgent character of the medical cases treated in the ED is inherent. Admission staff is trained specifically to assess urgency in every single case entering the premises and all processes regarding medical treatment, time and process management are based on these assessments [23]. These methods are well established and researched, aiding in providing patient safety and improving outcomes [25]. In order to function, the system must ignore the actual complexity of the patients admitted and reduce them into a manageable medical entity with one level of urgency [26].

There is already data indirectly proving the impact GPs have on acute medicine. In a recent study, half of the patients who used a telemedicine consultation to be advised on medical issues during weekday office hours stated that they would have gone straight to the ED if they had not received medical attendance this way. Interestingly, only 5% of these patients were finally advised to visit an ED [27]. This suggests that the work of GPs in the field of acute medicine in the primary care sector might have a significant impact on the patient load of EDs [28]. To what degree the acute medical problems dealt with by the GP`s are minor or severe ones, should be addressed in future research.

Another reason for a lack of data on the impact of GPs on acute medicine is the way GPs have to document their cases using code systems that do not include levels of urgency by design.

Coding systems like the ICD-10, ICPC2 or the SNOWMED-CT do not contain such an aspect [16, 18, 29]. Even more so, some of these coding systems are not even fit to code cases in General medicine. Data from the CONTENT study shows that in the most commonly coded ICD-10 diagnosis in GP practices, four different ICD codes are probably used to describe the same medical issue. This renders the system unsuitable for the undertaking of coding cases in GP practices. The extensive and impractical nature of the ICD-10 coding system, not fitting well to display the reasons for encounter in a GP setting, leads to it not being accepted enthusiastically by German GPs so far [16].

These differences must be considered when interpreting the findings and applicability of tools like ICPC codes in diverse settings. Highlighting these variations underlines the need for adaptable coding systems to accommodate international contexts [21].

The ICPC-2 surely is the better fit to display more accurately what the encounters in GP practices actually contain. But it would be far from wise to force yet another item of code onto the physicians to assess urgency in every medical case.

Additionally, the coding system found in the included publication allows for a real assessment of the kind of challenges GPs face daily. The structured documentation of the different aspects attributed to each of the listed problems with which a patient presents to the GP allows the multi-facetted consultation to be recorded [30]. A holistic assessment of any “case” in general medicine. Findings from this future research could help policy makers appreciate the role of the GP as the foundation of medical care not just in the chronic but finally also the acute sector.

Future research should do extensive surveys with an adapted version of the coding proforma identified in our systematic research. The large number of doctor-patient encounters happening in GP practices in most countries every day makes a representable result possible, even when only looking at the urgent cases of one day in the year.

### Strengths and limitations

A strength of this review is its structured approach. The comprehensive search strategy across several databases, coupled with the screening process facilitated by the web tool for systematic researches, ensured a broad capture of relevant studies.

Our review solely included publications in English or German making it not possible to have identified a study regarding a validated instrument for measuring urgency in medical cases in GP practices published in a different language.

## Conclusion

There is no readily available tool to simply assess urgent cases in primary care. The identified instrument to measure complexity in doctor-patient encounters will be used - with some adaptions to assess levels of urgency - to evaluate the contributions of GP`s in acute care making the benefits of holistic care visible.

## Electronic supplementary material

Below is the link to the electronic supplementary material.

Supplementary Material 1

## Data Availability

No datasets were generated or analysed during the current study.

## Abbreviations

ED: Emergency Department
GP: General Practitioner
ICD: International Statistical Classification of Diseases and Related Health Problems
ICPC: International Classification of Primary Care
LTUC: Life-threatening urgent cases
NLTUC: Non-life-threatening urgent cases
PRISMA: Preferred Reporting Items for Systematic Reviews and Meta-Analyses
RFE: Reason for Encounter
WICC: WONCA International Classification Committee
WONCA: World Organization of Family Doctors
