# Supplementary material for: An instrument as an action against the blind spot of acute medical care in general practice - a systematic review
Source: BMC Prim Care. 2025 Mar 8;26:67. doi: 10.1186/s12875-025-02749-6 (PMC11889748; doi:10.1186/s12875-025-02749-6)
Supplement: Supplementary file 1 — Supplementary Material 1 [file 12875_2025_2749_MOESM1_ESM.docx]

# Attachments:

## Attachment 1: Search string

(("Clinical Decision Making" OR "Decision-Making, Clinical" OR "Medical Decision-Making" OR "Decision-Making, Medical" OR "Medical Decision Making" OR "acute medical emergencies" OR "acute medical emergency" OR "urgent medical situations" OR "emergency medical care" OR "emergency interventions" OR "emergency healthcare" OR "trauma care" OR "immediate care" OR "urgent medical attention" OR "emergency therapy" OR "Emergency treatment" OR "Emergency Medical Services" OR "After-Hours primary Care" OR "emergency and out-of-hours care" OR "out-of-hours primary health care" OR "subacute care" OR "acute care" OR "subacute care") AND

("General Practice" OR "primary care" OR "Primary Health Care" OR "family medicine" OR "primary healthcare" OR "general practitioner services" OR "community medicine" OR "general medical practice" OR "family practice" OR "Ambulatory care" OR "outpatient care" OR "comprehensive health care" OR "Ambulatory care facility" OR "Outpatient health services" OR "Outpatient care" OR "Ambulance services" OR "Physicians, primary care" OR "Primary care offices") AND

("tool" OR "Health services research" OR "Routinely collected health data" OR "Classification" OR "Scoring system" OR "Clinical practice guidelines" OR "Clinical decision rules" OR “Weights and measures”) AND

(psychometric* OR "psychometric quality criteria" OR "instrument" OR "assessment tool" OR "measure" OR "questionnaire" OR "Health Surveys"))

##
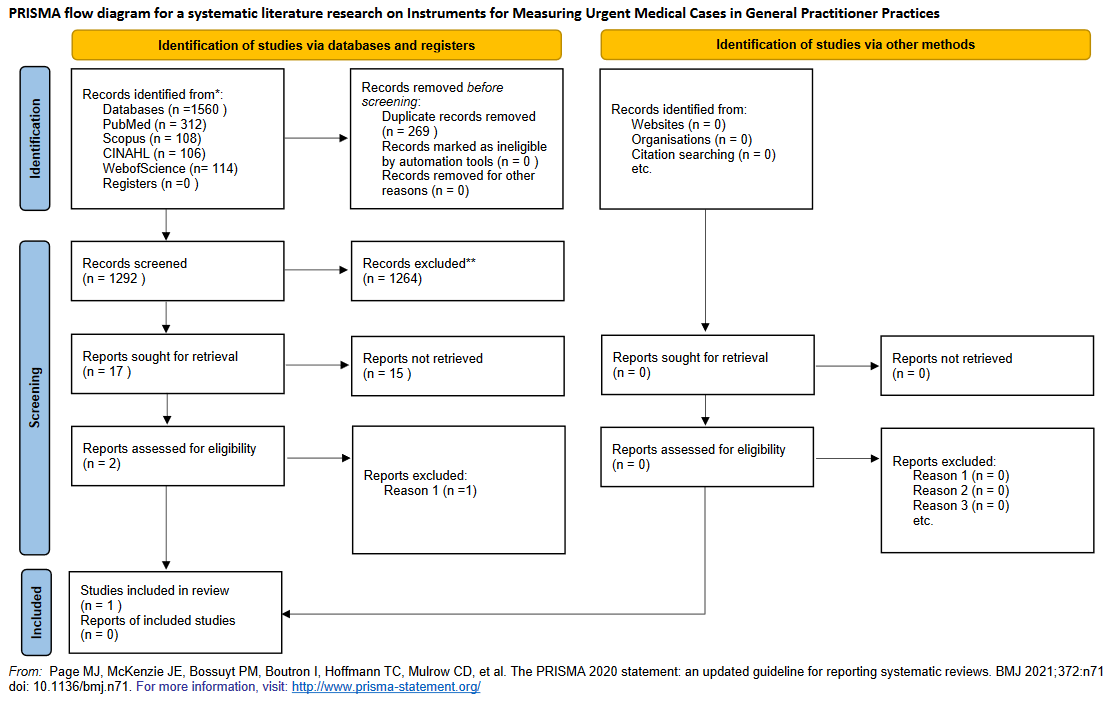
Attachment 2: PRISMA Flow Chart
